# 2D Zinc-Based Metal–Organic Complexes Derived N-Doped Porous Carbon Nanosheets as Durable Air Cathode for Rechargeable Zn–Air Batteries

**DOI:** 10.3390/polym14132581

**Published:** 2022-06-25

**Authors:** Peng Jia, Jiawei Zhang, Guangmei Xia, Zhenjiang Yu, Jiazhen Sun, Xingxiang Ji

**Affiliations:** State Key Laboratory of Biobased Material and Green Papermaking, Key Laboratory of Pulp and Paper Science & Technology of Ministry of Education/Shandong Province, Faculty of Light Industry, Qilu University of Technology (Shandong Academy of Sciences), Jinan 250353, China; skl_zhangjiawei@163.com (J.Z.); gmxia@qlu.edu.cn (G.X.); yuzhenjiang@hit.edu.cn (Z.Y.); jiazhensun@qlu.edu.cn (J.S.)

**Keywords:** metal–organic complexes, porous carbon nanosheets, oxygen reduction reaction, Zn–air batteries, N-doping, vacancy defects

## Abstract

The defect and N-doping engineering are critical to developing the highly efficient metal-free electrocatalysts for oxygen reduction reaction (ORR), mainly because they can efficiently regulate the geometric/electronic structures and sur-/interface properties of the carbon matrix. Herein, we provide a facile and scalable strategy for the large-scale synthesis of N-doped porous carbon nanosheets (NPCNs) with hierarchical pore structure, only involving solvothermal and pyrolysis processes. Additionally, the turnover frequency of ORR (TOF_ORR_) was calculated by taking into account the electron-transfer number (*n*). Benefiting from the trimodal pore structures, high specific surface area, a higher pore volume, high-ratio mesopores, massive vacancies/long-range structural defects, and high-content pyridinic-N (~2.1%), the NPCNs-1000 shows an excellent ORR activity (1600 rpm, *j*_s_ = ~5.99 mA cm^−2^), a selectivity to four-electron ORR (~100%) and a superior stability in both the three-electrode tests (CP test for 7500 s at 0.8 V, Δ*j*_s_ = ~0.58 mA cm^−2^) and Zn–Air battery (a negligible loss of 0.08 V within 265 h). Besides, the experimental results indicate that the enhancement of ORR activity mainly originates from the defects and pyridinic-N. More significantly, this work is expected to realize green and efficient energy storage and conversion along with the carbon peaking and carbon neutrality goals.

## 1. Introduction

In recent years, the serious exhaustion of fossil fuels and the increasing exhaustion of non-renewable energy have aroused widespread concerns about the energy crises and environmental problems. Hence, it is of great significance to develop clean and renewable energy storage and conversion devices [1,2,3,4,5,6,7]. Among the devices, zinc−air batteries (ZABs) are recognized as a promising candidate because of their low-cost, high safety, environmental friendliness, high theoretical energy density (1086 Wh kg^−1^), and high power density [8,9]. However, the oxygen reduction reaction (ORR) on the cathode acts as one key chemical reaction in the ZABs, which is subject to sluggish kinetics and high overpotential [10,11]. Thus, the highly efficient and stable catalysts were required to reduce overpotential and accelerate the ORR rate throughout the service time. Among all ORR electrocatalysts, though the noble metal-based electrocatalysts (Pt/C, Ir/C, RuO_2_, IrO_2_, et al.) derive the most advanced ORR activities, the high-cost, poor stability, and worse long-term durability enormously restrict their in-depth development and commercial applications [12,13,14]. Therefore, numerous investigations have been carried out to design and synthetize the non-noble metal/metal-free electrocatalysts with comparable ORR activity.

Metal-free nanocarbons doped with heteroatoms (such as N, O, P, S, B, and F) have been widely regarded as the most promising ORR electrocatalysts because of their superior activity, excellent durability, tunable geometric/electronic structures, and sur-/interface properties, and cost-effectiveness [15,16,17,18,19,20]. Among them, the N-doped nanocarbons are the most widely studied because they can activate the ORR process by providing the donor electrons to adjacent carbon atoms [21,22]. The N-doping (especially pyridinic-N) induced both electron transfer and electroneutrality break, which modify the charge/spin distributions in the nanocarbons and are mainly responsible for the remarkable ORR activity [23,24]. To our knowledge, the N-doping metal-free carbon materials are commonly provided that the electron-transfer number is lower than 3.93 [25,26]. Additionally, the intrinsic defects (such as armchair and zigzag edges, vacancies, and voids) in nanocarbons also can enhance ORR activity [27,28]. Furthermore, the construction of a hierarchical pore structure can improve the density of electroactive sites and accelerate ORR kinetics [29,30,31]. Specifically, the self-assembly and stacking of carbon nanosheets (CNSs) can form the hierarchical pore structure, and the nanoholes in the basal plane of CNSs can significantly increase active sites and bring additional lattice edges, which are more easily doped with nitrogen (N) to form the edged pyridinic-N and pyrrolic-N. Recent research has indicated that the combination between defects and N-doping in the CNSs can derive more ORR electroactive sites [32,33]. However, it still lacks a facile and scalable synthetic methodology for synergistically optimizing the defects and pyridinic-N in the CNSs.

In light of this, we developed a facile and scalable strategy for the commercial fabrication of the defect-enriched N-doped porous carbon nanosheets (NPCNSs) with the turbostratic graphite-like structure via pyrolyzing the solvothermally synthesized 2D zinc-based metal−organic complexes (Zn-MOCs). The Zn-MOCs nanosheets for subsequent pyrolysis were synthesized at the optimal adding amount of 4-nitro phthalic acid (NPA, 200 mg). After the pyrolysis, all NPCNSs were directly used without further treatment. The as-prepared NPCNSs derive the trimodal pore structures, high specific surface area (*S*_BET_), a higher pore volume (*V*_total_) high-ratio mesopores (*V*_meso_/*V*_tota_), massive vacancies/long-range structural defects, and high N-doping level. Such attractive merits endow NPCNSs with a short transport path and abundant active sites, further resulting in excellent ORR activity and kinetics. Remarkably, the assembled Zn–air battery with NPCNSs-1000 exhibits optimal performance.

## 2. Materials and Methods

### 2.1. Chemicals and Materials

Zinc nitrate hexahydrate (Zn(NO_3_)_2_·6H_2_O), 4-nitro phthalic acid (NPA), nitrilotriacetic acid (NTA), *N*,*N*-2-methylformamide (DMF), absolute alcohol, potassium hydroxide (KOH), and zinc acetate (Zn(C_2_H_3_O_2_)_2_) were obtained from Sinopharm Chemical Reagent Co., Ltd. (Beijing, China). Both commercial waterproof carbon paper with a microporous layer (Hesen HCP135) and Pt/C (20 wt.% Pt on Vulcan XC-72) and noble metal catalyst were obtained from Shanghai Hesen Electric Co., Ltd. (Shanghai, China). Zinc foils (99.994%, 0.1 mm) were purchased from Alfa Aesar (Shanghai, China). Nickel foams were obtained from Shenzhen Lvchuang Electronic Technology Co., Ltd. (Shenzhen, China). Nafion (5.0 wt.%) solution was purchased from DuPont Co. (Wilmington, NC, USA.). All the chemicals were directly used as received without further purification, while the zinc foil was polished before use. The deionized water (18.25 Ω cm) was used as a solvent for all aqueous solutions.

### 2.2. Synthesis of Zn-MOCs

Two-dimensional (2D) Zn-MOCs were synthesized via a solvothermal method. In a typical synthesis of Zn-MOCs-200, 450 mg of Zn(NO_3_)_2_·6H_2_O, 200 mg of NPA, and 200 mg of NTA were firstly dissolved in 30 mL of DMF under electromagnetic stirring to form a homogeneous solution. Subsequently, the above solution was transferred and sealed in a 50 mL Teflon-lined stainless-steel autoclave to solvothermally react at 150 °C for 8 h. After cooling down the autoclave to ambient temperature, the resulting precipitate was collected by vacuum suction filtration and washed five times with absolute alcohol. Finally, the white Zn-MOCs-200 precursor was received after being dried at 80 °C for 24 h. For comparative studies, other precursors also were synthesized by only changing the adding amount of NTA to 0, 50, 100, 150, and 300 mg and named Zn-MOCs-*x* (*x* = 0, 50, 100, 150, and 300: values of NTA in milligram).

### 2.3. Preparation of NPCNSs

**Figure 1:**
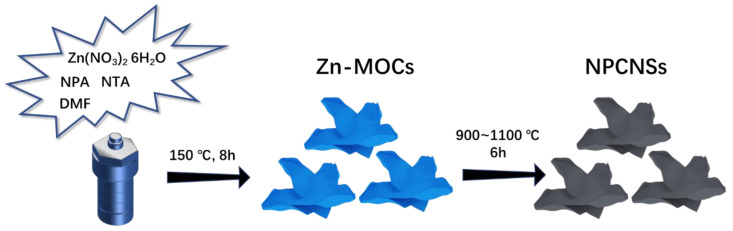
Schematic illustration for the fabrication process of NPCNSs.

Preferably, all NPCNSs were prepared via a one-step calcination/self-activation process of Zn-MOCs-200 precursor, as schematically illustrated in Figure 1. In a typical procedure of NPCNSs-1000, the Zn-MOCs-200 precursor was heated up to 1000 °C at a ramping rate of 2.0 °C/min and maintained for 6 h under a flow of argon. After cooling down the tubular furnace to ambient temperature, the black NPCNSs-1000 product was directly obtained without further purification. In order to achieve some insights in terms of the electrocatalytic activity, other products also were prepared by only changing the target temperature to 900 °C and 1100 °C and named NPCNSs-*y* (*y* = 900 and 1100: values of target temperature in centigrade).

### 2.4. Material Characterization

The microstructures and morphologies of precursors and catalysts were characterized by field-emission scanning electron microscopy (FESEM, FEI QUANTA FEG 250) and a high-resolution transmission electron microscopy (HR-TEM, JEOL JEM-2100F). The compositions of catalysts were detected by an X-ray energy spectrometer attached to a TEM. The phase constitutions of precursors and catalysts were determined by collecting X-ray diffraction (XRD) patterns on a Bruker D8 Advance diffractometer (λ = 0.15406 nm). Raman spectra of catalysts were collected on a T64000 Raman spectrometer (λ_0_ = 514.5 nm) to evaluate the graphitization degree and structural size. The nitrogen ad-/desorption isotherms were collected on a TriStar II 3020 surface area analyzer to analyze the pore structure, and the micro-/mesoporous diameter distribution data were calculated by the classic nonlocal density function theory (NLDFT) and Barrett−Joyner−Halenda (BJH) methods, respectively. X-ray photoelectron spectroscopy (XPS) spectra were collected on a VGESCALAB MKII spectrometer to analyze the element valences and composition on the surface of each catalyst. The bulk electrical conductivities of all NPCNSs were determined via a four-probe system equipped with DC current-reversal equipment [34].

### 2.5. Electrochemical Evaluation of Catalyst

The electrochemical activity of the catalyst was evaluated with cyclic voltammetry (CV), linear sweep voltammetry (LSV), and chronopotentiometry (CP) technologies on a rotating electrode system (Pine Instrument Co., Grove City, PA, USA) coupled with a CHI760E electrochemical workstation (Shanghai Chen Hua Instrument Co., Ltd., Shanghai, China). All electrochemical measurements were conducted in a three-electrode system: a counter electrode of the platinum plate, a reference electrode of Ag/AgCl (saturated KCl, 0.197 V vs. SHE), and a working electrode of glassy carbon rotating disk electrode (RDE, 5 mm in diameter) loaded with a catalyst or a rotating ring-disk electrode (RRDE, 7.5 mm in the outer diameter of the platinum ring, 6.5 mm in the inner diameter of the platinum ring, 5 mm in the diameter of glassy carbon disk loaded with the catalyst). In order to ensure that RDE and RRDE were evenly loaded by the catalyst, the catalyst ink was firstly prepared by introducing 4 mg of catalyst and 50 μL of Nafion solution (5.0 wt.%) into 950 μL of water-isopropanol solution mixed with 450 μL of water and 500 μL of isopropanol and sonicating the above mixture for 2 h. The same recipe was adopted to prepare the Pt/C (20 wt.%) catalyst ink. Afterwards, 20 μL of catalyst ink was coated onto RDE or RRDE and dried in air for the ORR test, obtaining a catalyst loading of 0.4 mg cm^−2^. The loading of Pt/C (20 wt.%) is 0.2 mg cm^−2^.

O_2_ (99.999%) was bubbled through the electrolyte solution (0.1 M KOH) for 30 min for saturation before the ORR test, and a flow of O_2_ was maintained over the electrolyte during the ORR test. For RDE measurement, CV curves were measured at a scanning rate of 10 mV s^−1^ and a rotating rate of 1600 rpm, and LSV curves were collected at a scanning rate of 10 mV s^−1^ and various rotating rates of 400, 625, 900, 1225, 1600, 2025, and 2500 rpm. For comparison, CV curves also were measured in an Ar (99.99%)-saturated electrolyte. For RRDE measurements, the operating potential of the Pt ring was set as 0.5 V vs. Ag/AgCl, and LSV curves were collected at a scanning rate of 10 mV s^−1^ and a rotating rate of 1600 rpm in an oxygen-saturated 0.1 M KOH solution. Furthermore, CV curves were collected at different scanning rates of 5, 10, 20, 30, 40, 50, 60, 70, 80, 90, and 100 mV s^−1^ in a potential window of 100 mV centered at 1.116 V vs. RHE to evaluate the electrochemically active surface area (ECSA) of catalyst. Besides, the electrochemical impedance spectroscopy (EIS) spectra were measured to evaluate the resistances for the ORR reaction.

All potentials were corrected with an ohmic drop of ~32.0 Ω, namely *iR* compensation. Subsequently, all measured potentials were converted to the reversible hydrogen electrode (RHE) scale according to the Nernst equation (Equation (1)) [35].

(1) $$E_{\mathrm{RHE}}=E_{\mathrm{Ag}/\mathrm{AgCl}}+0.0591\times \mathrm{pH}+0.197\;V$$

where *E*_RHE_ and *E*_Ag/AgCl_ represent the potentials vs. RHE and Ag/AgCl electrode, respectively. pH is ~13.0 for 0.1 M KOH. Based on LSV data, the Tafel slope (*b*, mV dec^−1^) was calculated according to the Tafel equation (Equation (2)) [36].

(2) η=a+blog(j)

where *η* represents the overpotential (mV), and *j* stands for the current density (mA cm^−2^).

Based on the RRDE approach, the yield of hydroperoxyl anion (${\mathrm{HO}}_{2}^{-}$%) and total electron-transfer number (*n*) were determined according to Equations (3) and (4), respectively [37,38].

(3) $${\mathrm{HO}}_{2}^{-}\;\%=\frac{2i_{r}}{i_{r}+Ni_{d}}\times 100\%$$

(4) $$n=\frac{4Ni_{d}}{i_{r}+Ni_{d}}$$

where *i_r_* and *i_d_* stand for the currents through the ring and disk electrodes, respectively. *N* represents the collection efficiency of RRDE, which was determined as 0.37. ECSAs (${\mathrm{cm}}_{\mathrm{ECSA}}^{2}$ ${\mathrm{cm}}_{\mathrm{GEO}}^{-2}$) were calculated according to Equation (5) [39].

(5) $$\mathrm{ECSA}=1000\times \frac{C_{dl}}{C_{s}}$$

where *C_dl_* stands for the electrochemical double-layer capacitance per geometric area (mF ${\mathrm{cm}}_{\mathrm{GEO}}^{-2}$). *C_s_* corresponds to the specific capacitance per ECSA, which is considered as a standard value of 40 μF ${\mathrm{cm}}_{\mathrm{ECSA}}^{-2}$ in this work.

### 2.6. Electrochemical Evaluation of Rechargeable Zn–Air Batteries

For traditional stack-type battery evaluation, zinc–air batteries were assembled with a cathodic current collector of cleaned and hot-pressed nickel foam, an anode of polished Zn foil, and a cathode of carbon paper loaded with catalyst (0.4 mg cm^−2^) in which the aforementioned catalyst ink was dripped onto carbon paper (1 cm × 1 cm). The electrolyte was in an aqueous solution containing 6.0 M KOH and 0.2 M Zn(C_2_H_3_O_2_)_2_, which ensured the reversible anodic electrochemical reactions. All Zn–air batteries were evaluated on a LAND CT2001A battery test system under the ambient atmosphere (~25 °C). The specific capacity of zinc–air batteries (*C*_ZAB_, mAh g^−1^) was calculated according to Equation (6) [40].

(6) $$C_{\mathrm{ZAB}}=\frac{i\times t}{m_{\mathrm{Zn}}}$$

where *i* stands for the current through zinc–air battery (mA), *t* represents the service time (h), and *m*_Zn_ corresponds to the mass of consumed zinc (g).

## 3. Results and Discussion

### 3.1. Microstructures of Zn-MOCs

**Figure 2:**
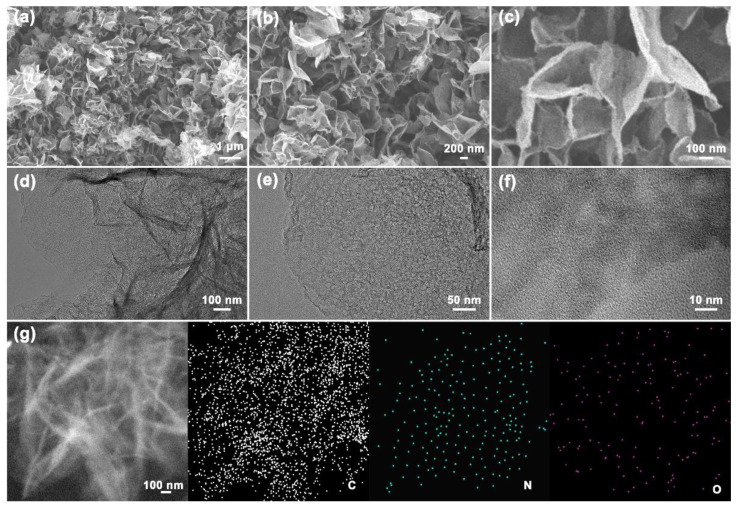
(**a**–**c**) SEM, (**d**,**e**) TEM, (**f**) HRTEM, and (**g**) STEM and corresponding the element mapping of C, N, and O for NPCNSs-1000.

In order to optimize the preparation process, the morphologies of all Zn-MOCs were observed and the SEM images are depicted in Figure 2. Only the Zn-MOCs-0 nanospheres form without the addition of NTA (Figure 2a). The nanosheets and nanospheres coexist in Zn-MOCs-50, Zn-MOCs-100, and Zn-MOCs-150 and the volume fraction of nanosheets raises with the increase in the adding amount of NTA (Figure 2b–d). Only the Zn-MOCs-200 and Zn-MOCs-300 nanosheets form by adding no less than 200 mg of NTA. However, the coarsened nanosheets appear when the adding amount of NTA is more than 200 mg. Thus, the addition of NTA is conducive to the formation of nanosheets and the optimum adding amount is 200 mg.

The phase compositions of all Zn-MOCs were determined from the XRD patterns, as shown in Figure S1. Apparently, there is only the ZnO phase (PDF#97-016-5002) in Zn-MOCs-0. ZnO and Zn-MOC (CCDC1444472) phases coexist in Zn-MOCs-50, Zn-MOCs-100 and Zn-MOCs-150 [41]. There is only the Zn-MOC phase in Zn-MOCs-200 and Zn-MOCs-300. Combined with the SEM results, it can be deduced that the nanosheets and nanospheres correspond to Zn-MOC and ZnO phases, respectively. Therefore, 2D Zn-MOC nanosheets can be synthetized at the optimum NTA adding an amount of 200 mg.

### 3.2. Microstructures and Physicochemical Properties of NPCNSs

The microstructure and composition distribution of NPCNSs-1000 were further analyzed from the SEM, TEM, HRTEM, STEM, and EDS mapping in Figure 2. The NPCNSs-1000 nanosheets assemble into the micron flowers (Figure 2a–c), which is beneficial for the increase in electrical conductivity. Additionally, the slit macro-/mesopores form in the micron flowers (Figure 2c,d). Plenty of meso-/micropores from the NPCNSs-1000 nanosheets can be found (Figure 2e,f). Hence, the trimodal pore structure forms in the NPCNSs-1000, which is beneficial for the shortening of the transport path of reactants and products and the increase in the specific surface area (*S*_BET_). The C, N, and O elements are evenly distributed in the NPCNSs-1000 nanosheets (Figure 2g), which is conducive to the efficient utilization of ORR active sites.

**Figure 3:**
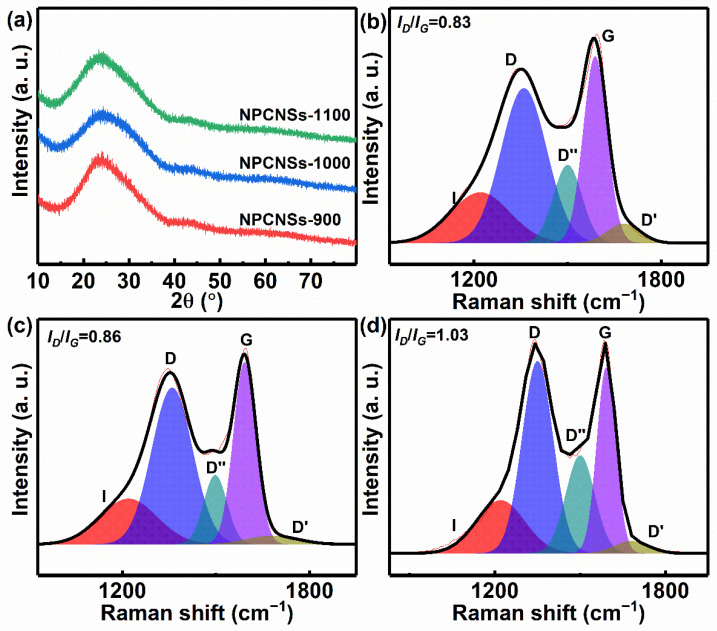
(**a**) XRD patterns of NPCNSs-*y* (*y* = 900, 1000, and 1100), Raman spectra of (**b**) NPCNSs-900, (**c**) NPCNSs-1000, and (**d**) NPCNSs-1100.

The XRD patterns of all the samples show one obvious broadening peak at ~22.0° and another weaker broadening peak at ~43.9°, which are assigned to (002) and (100) crystal planes of carbon (Figure 3a), indirectly indicating for the turbostratic graphite-like structure with the highly exposed (002) crystal planes. Additionally, no other peaks can be found from XRD patterns, indicating that zinc can be completely evaporated and removed from all samples above 900 °C. Furthermore, the interplanar spacing (*d*_002_), the crystallite size (*L_c_*), and the in-plane crystal size (*L_a_*) of all NPCNSs were determined based on the Bragg’s law (Equation (7)) and Debye–Scherrer equations (Equations (8) and (9)) [42].

(7) $$d_{00}{}_{2}=\frac{\lambda }{2\sin\theta_{002}}$$

(8) $$L_{c}=\frac{0.89\lambda }{\beta_{002}\cos\theta_{002}}$$

(9) $$L_{a}=\frac{1.84\lambda }{\beta_{100}\cos\theta_{100}}$$

where *β* corresponds to the full width at half maximum (FWHM) of diffraction peak in radian, and *θ* represents the Bragg angle in degree. All calculated values of the above structural parameters are summarized in Table S1.

The NPCNSs-1000 has a lower diffraction angle of ~25.00° for the (002) crystal plane than NPCNSs-900 (~25.06°) and NPCNSs-1100 (~25.60°), suggesting that the NPCNSs-1000 derives the highest *d*_002_ of ~0.356 nm (Table S1). Furthermore, the NPCNSs-1000 and NPCNSs-1100 have the smaller *L*_c_ (~0.59 and ~0.60 nm) and *L*_a_ (~2.67 and ~2.23 nm) than NPCNSs-900 (*L*_c_, ~0.66 nm; *L*_a_, ~2.80 nm), indicating for the more defect sites in NPCNSs-1000 and NPCNSs-1100 and benefiting for shortening the transport path of reactants and products and increasing the *S*_BET_. In order to further determine the intrinsic structure of all samples, the Raman spectra were collected and fitted with the following five Gaussian peaks; namely, five Raman modes (Figure 3b–d) [43,44,45].

- I-band (~1220 cm^−1^): *sp*^2^–*sp*^3^ bonds or stretching vibrations of C–C and C=C in polyene-like structures.
- D-band (1360 cm^−1^): *A*_1g_ phonons at the Dirac point (*K*-point) resulting from the breathing mode of hexatomic rings indicating the disordered carbons and defective graphitic structures, and the broadening of which demonstrates more short-range structural defects such as domain boundaries, edges, vacancies, and doped heteroatoms or more disordered sites.
- D″-band (~1500 cm^−1^): the amorphous carbon, and the intensity of which is inversely proportional to the crystallinity.
- G-band (~1590 cm^−1^): optical *E*_2g_ phonons at the Brillouin zone center (*Γ*-point) arising from in-plane stretching vibrations of *sp*^2^–*sp*^2^ bands, and the broadening of which suggests more disorder in the bond length and angle of *sp*^2^–*sp*^2^ bands or more *sp*^3^ carbon atoms.
- D′-band (~1680 cm^−1^): the intensity of which is positively correlated with the ratio in the density of states of graphite to disordered graphitic lattices.

The apparent D-band and D′-band peaks imply plenty of structural defects in all samples. With the increase in pyrolysis temperature from 900 °C to 1000/1100 °C, the FWHM of the D-band peak drops from ~172 to ~154/~128 cm^−1^ and the FWHM of the G-band peak descends from ~90 to ~85/~79 cm^−1^, indicating for the decrease in the short-range structural defects and more order in the short-range scale, respectively. The area percentage D’-band peak drops from ~3.69% to ~2.90%/~2.71%, implying a decrease in the disordered graphitic lattices. The area percentage D”-band peak raises from ~10.64% to ~12.75%/~18.48%, demonstrating a decrease in the crystallinity. With the increase in pyrolysis temperature from 900 °C to 1000/1100 °C, the *I_D_*/*I_G_* rises from 0.83 to 0.86/1.03, indicating more disorder/defects and a decrease in the graphitization degree. The bulk electrical conductivity of NPCNSs-1100 (~65.1 S cm^−1^) is higher than that of NPCNSs-1100 (~52.9 S cm^−1^) and NPCNSs-1100 (~ 60.6 S cm^−1^), indicating an increase in the bulk electrical conductivity. Additionally, *L*_a_ (nm) also was calculated based on the Tuinstra–Koenig equation (Equation (10)) [45].

(10) $$L_{a}=4.4\times {\left(\frac{I_{D}}{I_{G}}\right)}^{-1}$$

The *L*_a_ of NPCNSs-1100 (~2.23 nm) is smaller than that of NPCNSs-900 (~2.77 nm) and NPCNSs-1000 (~2.67 nm), which is in line with the XRD results, indicating that the more boundaries (namely long-range structural defects) appear in the turbostratic graphite-like structure with an increase in pyrolysis temperature from 900 °C to 1100 °C.

In a word, the total density of short-range structural defects drops benefiting from the increase in the conductivity and electron transmission rate, while the density of long-range structural defects raises with the increase in pyrolysis temperature from 900 °C to 1100 °C benefiting from providing more ORR active sites.

**Figure 4:**
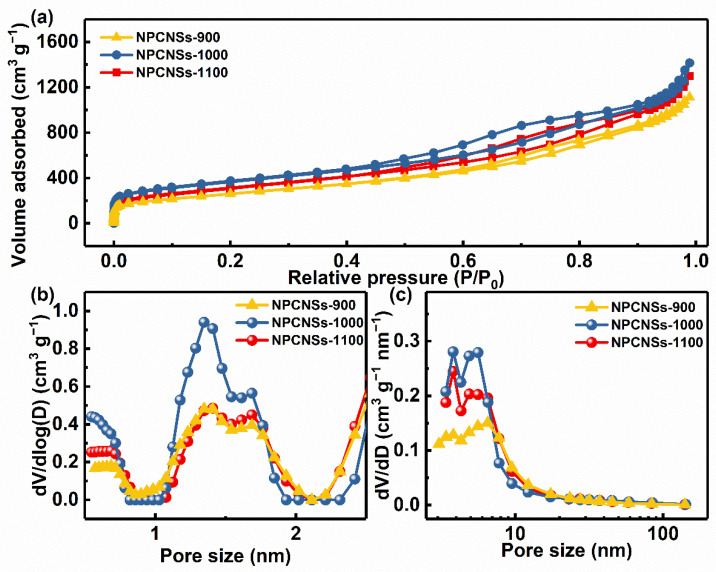
(**a**) N_2_ ad-/desorption isotherms, (**b**) microporous, and (**c**) mesoporous diameter distribution curves of NPCNSs-900, NPCNSs-1000, and NPCNSs-1100.

In order to quantitatively characterize the porous structure, the N_2_ ad-/desorption isotherms were measured, and the recorded data and analysis results are shown in Figure 4 and Table S2. As shown in Figure 4a, the NPCNSs-1000 obtains a larger adsorption capacity of ~1414.8 cm^3^ g^−1^ than NPCNSs-900 (~1113.1 cm^3^ g^−1^) and NPCNSs-1100 (~1299.4 cm^3^ g^−1^), demonstrating the NPCNSs-1000 has the higher *S*_BET_ (~1300.8 m^2^ g^−1^) and total pore volume (*V*_total_, ~2.2 cm^3^ g^−1^) than NPCNSs-900 (*S*_BET_, ~942.5 m^2^ g^−1^; *V*_total_, ~1.7 cm^3^ g^−1^) and NPCNSs-1100 (*S*_BET_, ~1129.7 m^2^ g^−1^; *V*_total_, ~2.0 cm^3^ g^−1^). According to the latest classifications of physisorption isotherms and hysteresis loops by the International Union of Pure and Applied Chemistry (IUPAC), NPCNSs-900, NPCNSs-1000, and NPCNSs-1100 show the integrations of the following typical characteristics [46,47].

- Extremely low relative pressure zone (P/P_0_ < 0.01): type I(a) isotherms with sharp adsorption for indicating lots of ultramicropores (*d* < 0.7 nm).
- Low relative pressure zone (0.01 < P/P_0_ < 0.15): type I(b) isotherms with discernible adsorption for demonstrating lots of supermicropores (0.7 < *d* < 2.0 nm).
- Medium relative pressure zone (0.15 < P/P_0_ < 0.9): type IV(a) isotherms and an H4-type hysteresis loops with a notable adsorption capacity for revealing plenty of mesopores.
- High relative pressure zone (0.9 < P/P_0_ < 1.0): type II isotherms with an obvious upward tendency to adsorption capacity for implying the abundant macropores.

The aforementioned characteristics show that all samples have the trimodal pore structure in which micropores, mesopores, and macropores coexist, which accords with the above SEM, TEM, and HRTEM results. The hierarchical porous structures also can be evidenced by microporous and mesoporous diameter distribution curves (Figure 4b,c). All samples have similar micro-/mesopore diameter distribution curves due to the same formation mechanism of pores, namely the volatilization of zinc. Additionally, the NPCNSs-1000 has more micropores (*V*_micro_, ~1.8 cm^3^ g^−1^) and mesopores (*V*_meso_, ~0.4 cm^3^ g^−1^) than NPCNSs-900 (*V*_micro_, ~1.5 cm^3^ g^−1^; *V*_meso_, ~0.2 cm^3^ g^−1^) and NPCNSs-1100 (*V*_micro_, ~1.7 cm^3^ g^−1^; *V*_meso_, ~0.3 cm^3^ g^−1^), indicating that the moderate temperature of 1000 °C is more conducive to the formation of pores. The lower temperature of 900 °C is detrimental to the rapid volatilization of zinc, which limits the formation of pores. The sample is easy to sinter at a higher temperature of 1100 °C, which leads to the reduction in pore volume and diameter. The appropriate pyrolysis temperature also can be evidenced by the fact that the average pore diameter (APD, ~7.3 nm) of NPCNSs-1000 is larger than that of NPCNSs-900 (APD, ~6.7 nm) and NPCNSs-1100 (APD, ~7.1 nm), as shown in Table S2. Besides, the NPCNSs-1000 derives ~18.1% of the porosity from mesopores, which is higher than that of NPCNSs-900 (~11.8%) and NPCNSs-1100 (~15.0%). As widely known, the mesopores are in favor of the penetration of electrolytes into the inner micropores which is beneficial for improving the ECSA and the transport of reactant (O_2_) and products (${\mathrm{HO}}_{2}^{-}$ and OH^−^) and accelerating the ORR kinetics, particularly at high ORR reaction rates. Thus, the better collocation of mesopores and micropores for NPCNSs-1000 is beneficial for improving ORR activity and kinetics.

**Figure 5:**
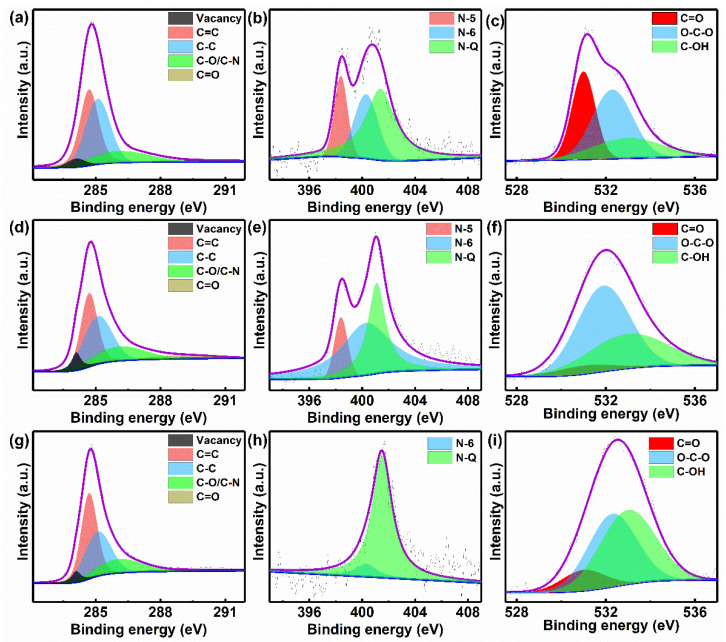
((**a**,**d**,**g**) C 1s, (**b**,**e**,**h**) N 1s and (**c,f,i**) O 1s of (**a**–**c**) NPCNSs-900, (**d**–**f**) NPCNSs-1000, and (**g**–**i**) NPCNSs-1100.

In order to quantitatively analyze the chemical compositions and states on the surface of the NPCNSs-900, NPCNSs-1000, and NPCNSs-1100, XPS spectra were collected and fitted, as shown in Figure 5. The high-resolution C 1s spectra are typically divided into the five peaks (Figure 5a,d,g), corresponding to C-I (~284.1 eV, vacancy), C-II (~284.7 eV, C=C), C-III (~285.1 eV, C–C), C-IV (~286.1 eV, C–O/C–N), and C–V (~289.0 eV, C=O) species [48]. In regard to the N 1s spectra, N-I (~398.4 eV, N–5, pyrrolic-N), N-II (~400.3 eV, N–6, pyridinic-N), and N-III (~401.4 eV, N–Q, graphitic-N) peaks are depicted in Figure 5b,e,h. For the O 1s spectra, O-I (~531.0 eV, O=C), O-II (~532.3 eV, C–O–C), and O-III (~533.0 eV, C–OH) are shown in Figure 5c,f,i.

The XPS analysis results are summarized in Table S3. The surface compositions are as follows: NPCNSs-900 (C, ~90.8%; N, ~4.5%; O, ~4.7%), NPCNSs-1000 (C, ~91.7%; N, ~3.9%; O, ~4.4%), and NPCNSs-1100 (C, ~95.7%; N, ~1.7%; O, ~2.6%), indicating the decrease in the heteroatom contents (N or O element) with the increase in pyrolysis temperature. The area percentage of the C-I peak (~5.8%) for NPCNSs-1000 is larger than that of NPCNSs-900 (~3.2%) and NPCNSs-1100 (~4.2%), implying the most point defects resulting from carbon vacancies. The result also indicates that the volatilization of Zn can effectively improve the content of vacancies at the appropriate temperature. More vacancies benefit from the increase in the ECSA. It is worth noting that this result does not contradict the fact that the total density of short-range structural defects drops with the increase in the pyrolysis temperature, due to the changing trend in the density of other short-range structural defects (such as the doping heteroatom), which is the same as that of the total density of short-range structural defects. The N species are as follows: NPCNSs-900 (N–5, ~0.8%; N–6, ~1.2%; N–Q, ~2.5%), NPCNSs-1000 (N–5, ~0.5%; N–6, ~2.1%; N–Q, ~1.3%) and NPCNSs-1100 (N–6, ~0.2%; ~N–Q, 1.5%). The higher contents of pyrrolic-N (N–5) and pyridinic-N (N–6) are beneficial for the ORR activity.

### 3.3. ORR Performances of NPCNSs

**Figure 6:**
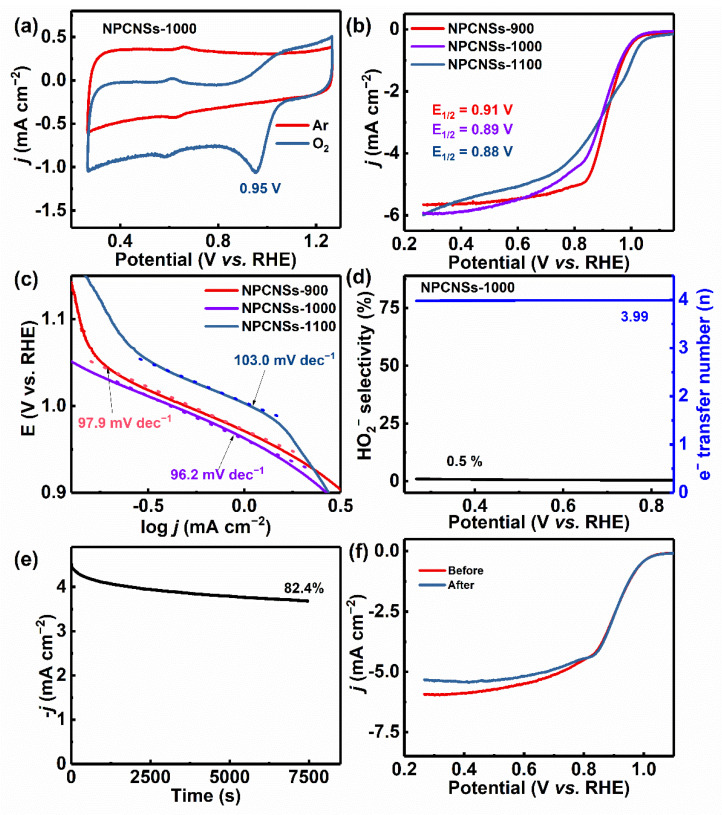
(**a**) CV curves of NPCNSs-1000 in the Ar-saturated and O_2_-saturated 0.1 M KOH solutions, (**b**) ORR LSV curves at 1600 rpm for and (**c**) ORR Tafel curves for NPCNSs-*y* (*y* = 900, 1000, and 1100), (**d**) ${\mathrm{HO}}_{2}^{-}$ yield and electron-transfer number (*n*), (**e**) CP curves at 0.8 V vs. RHE, and (**f**) LSV curves before and after CP test for NPCNSs-1000.

The ORR performances of NPCNSs-*y* (*y* = 900, 1000, and 1100) are presented in Figure 6. The CV curves of all samples have obvious reduction peaks in O_2_-saturated electrolyte solution but not in Ar-saturated electrolyte solution (Figure 6a and Figure S2), preliminarily indicating the existence of ORR activity in all samples. Additionally, the cathodic peak potential of NPCNSs-1000 (~0.95 V) is the same as that of NPCNSs-900 (~0.95 V) and even higher than that of NPCNSs-1100 (~0.88 V). Furthermore, the ORR catalytic activities of all samples were measured with LSV technology in 0.1 M KOH. As shown in Figure 6b, NPCNSs-1000 has a larger limited current density (*j*_s_ = ~5.99 mA cm^−2^) than NPCNSs-900 (~5.65 mA cm^−2^) and NPCNSs-1100 (~5.92 mA cm^−2^), and the half-wave potential of NPCNSs-1000 (*E*_1/2_, ~0.89 V) is comparable to that of NPCNSs-900 (~0.91 V) and NPCNSs-1100 (~0.88 V). Moreover, the ORR kinetics were studied via the Tafel curves. As depicted in Figure 6c, the Tafel slope of NPCNSs-1000 (*b*, ~96.2 mV dec^−1^) is lower than that of NPCNSs-900 (~97.9 mV dec^−1^) and NPCNSs-1100 (~103.0 mV dec^−1^), indicating the most favorable ORR kinetics of NPCNSs-1000. Hence, the vacancy defects and N-doping can efficiently enhance ORR activity and accelerate ORR kinetics.

Except for the ORR activity, a high ORR selectivity is one of the prerequisites towards practical applications. The LSV curves at the different scanning rates and RRDE curves were measured, and the ${\mathrm{HO}}_{2}^{-}$ yield and electron-transfer number (*n*) were calculated to evaluate the ORR selectivities of samples (Figure 6d, Figures S3 and S4). As depicted in Figure 6d and Figure S4c,f, the electron-transfer number of oxygen reduction of NPCNSs-1000 is ~3.99 in a wider potential range from 0.85 to 0.27 V vs. RHE, which is higher than that of NPCNSs-900 (3.55–3.91) and NPCNSs-1100 (3.45–3.65), implying that NPCNSs-1000 exhibits ~100% selectivity for four-electron ORR. Take 0.42 V for example, the electron-transfer numbers are ~3.99 (NPCNSs-1000), ~3.63 (NPCNSs-900), and ~3.46 (NPCNSs-1100), corresponding to ${\mathrm{HO}}_{2}^{-}$ yields of ~0.5%, ~18.6%, and ~27.1%, respectively.

The Nyquist plots were used to evaluate the charge transfer resistances (R_ct_), as shown in Figure S6a. In order to make the quantitative analysis, the equivalent circuit diagram was drawn in Figure S6a and the corresponding fitted impedance values for Nyquist plots were summarized in Table S4. It can be found from Table S4 that the R_ct_ of NPCNSs-1000 (~10.6 Ω) is smaller than those of NPCNSs-900 (~20.3 Ω) and NPCNSs-1100 (~14.5 Ω). Besides, the Bode plots were used to evaluate the resistances for adsorption and migration of O_2_ molecules (Z_cdl_). As shown in Figure S6b, the peaks in a frequency range of 0.01–10 Hz involve the adsorption and migration of O_2_ molecules. The peak frequency for NPCNSs-1000 (~0.121 Hz) is higher than those of NPCNSs-900 (~0.038 Hz) and NPCNSs-1100 (~0.046 Hz), indicating that the Z_cdl_ of NPCNSs-1000 is smaller than those of NPCNSs-900 and NPCNSs-1100. Therefore, the basic steps involving the adsorption and migration of O_2_ molecules and the charge transfer are more beneficial for NPCNSs-1000.

Comprehensively considering the activity and selectivity, the NPCNSs-1000 has more application value towards four-electron ORR catalyst among all samples. Besides, an ORR high stability also is one of the prerequisites towards practical applications. Hence, the CP curve and LSV curves before and after the CP test were measured to evaluate the stability of NPCNSs-1000 (Figure 6e,f). The current retention is ~82.4% after 7500 s ($r_{0.8}^{7500}$) at a low overpotential of 0.8 V vs. RHE (Figure 6e), indicating the excellent ORR stability of NPCNSs-1000. This is further determined by the fact that the limited current density decreases from 5.99 to 5.41 mA cm^−2^ (Δ*j*_s_ = ~0.58 mA cm^−2^) after the CP test (Figure 6f). Besides, the peak frequency is still at ~0.121 Hz after the CP test (Figure S7), indicating the good ORR stability of NPCNSs-1000. In summary, the NPCNSs-1000 derives the optimum activity, four-electron ORR selectivity, and stability and shows the high application potential in the four-electron reduction.

The intrinsic catalytic activities of the catalysts were further evaluated. Based on the collected CV curves at different scanning rates in a non-Faradaic region (Figure S5a–c), the values of C_dl_ for all samples were estimated via plotting the half difference between anodic (*j*_a_) and cathodic (*j*_c_) current densities at 1.116 V vs. RHE, namely Δ*j* = 0.5(*j*_a_ − *j*_c_), against the scanning rate *v* (mV/s), and which are equal to the slopes of linear fitting curves for the plotted scatter diagram (Figure S5d) [49], i.e., 11.8 (NPCNSs-900), 14.1 (NPCNSs-1000) and 5.9 mF ${\mathrm{cm}}_{\mathrm{GEO}}^{-2}$ (NPCNSs-1100). Obviously, NPCNSs-1000 has a higher ECSA (~352.5 ${\mathrm{cm}}_{\mathrm{ECSA}}^{2}$ ${\mathrm{cm}}_{\mathrm{GEO}}^{-2}$) than NPCNSs-900 (~295.0 ${\mathrm{cm}}_{\mathrm{ECSA}}^{2}$ ${\mathrm{cm}}_{\mathrm{GEO}}^{-2}$) and NPCNSs-1100 (~147.5 ${\mathrm{cm}}_{\mathrm{ECSA}}^{2}$ ${\mathrm{cm}}_{\mathrm{GEO}}^{-2}$), indicating most electrochemically active sites on the surface of NPCNSs-1000. The ECSA is correlated with the *S*_BET_, the densities of vacancy and long-range structural defects indicate that more vacancy and long-range structural defects benefit from the increase in the ECSA and ORR activity.

In order to evaluate the intrinsic catalytic activity of the catalyst, the turnover frequency (TOF, h^−1^) for ORR was calculated based on Equation (11) [50].

(11) $${\mathrm{TOF}}_{\mathrm{ORR}}=3600\times \frac{n_{O_{2}}}{n_{\mathrm{site}}}$$

where the total oxygen turnovers per geometric area per second (*n*_$O_{2}$_, molecules ${\mathrm{cm}}_{\mathrm{GEO}}^{-2}$ s^−1^) can be calculated from the current density (*j*_ORR_, mA ${\mathrm{cm}}_{\mathrm{GEO}}^{-2}$) derived from the LSV curve according to Equation (12).

(12) $$\begin{array}{ll} n_{O_{2}} & =\left|j_{\mathrm{ORR}}\right|\times \frac{\mathrm{mA}}{{\mathrm{cm}}_{\mathrm{GEO}}^{2}}\times \frac{1\;C\cdot s^{-1}}{1000\;\mathrm{mA}}\times \frac{1\;\mathrm{mol}\;e^{-1}}{96485.3\;C}\times \frac{1{\;\mathrm{mol}\;O}_{2}}{4\;\mathrm{mol}\;e^{-1}}\times \frac{6.022\times 10^{23}{\;O}_{2}\;\mathrm{molecules}}{1{\;\mathrm{mol}\;O}_{2}}\times \frac{6-n}{2}\\ & =1.56\times 10^{15}\times \left|j_{\mathrm{ORR}}\right|\times \frac{6-n}{2}\times \frac{O_{2}\;\mathrm{molecules}\cdot s^{-1}\;}{{\mathrm{cm}}_{\mathrm{GEO}}^{2}} \end{array}$$

In this work, the ORR active sites are designated as the carbon atoms adjacent to the pyridinic-N and pyrrolic-N. Additionally, all catalysts show the highly exposed (002) crystal planes. Hence, the total active sites per geometric area (*n*_site_, atoms ${\mathrm{cm}}_{\mathrm{GEO}}^{-2}$) can be calculated according to Equation (13).

(13) $$n_{\mathrm{site}}=\frac{\mathrm{ECSA}}{A_{0}}\times \frac{\mathrm{atoms}}{{\mathrm{cm}}_{\mathrm{GEO}}^{2}}$$

where A_0_ is the electrochemically active surface area per active site, which is approximately equal to the average cross-sectional area of carbon atoms on (002) crystal planes (2.62 × 10^−16^  ${\mathrm{cm}}_{\mathrm{ECSA}}^{2}$ atom^−^^1^). Finally, the values of TOF_ORR_ at 0.42 V vs. RHE can be calculated according to Equation (14), i.e., 33.4 h^−1^ (NPCNSs-900), 23.0 h^−1^ (NPCNSs-1000), and 74.5 h^−1^ (NPCNSs-1100), which is comparable and even superior to some recently reported catalysts (Table S5) [50,51,52,53,54,55]. In a word, although the NPCNSs-1000 has the highest ORR activity (5.99 mA cm^−2^), the NPCNSs-1100 derives the highest intrinsic activity. Based on the negative correlation between the intrinsic activity and ECSA, this may be due to the competition among ORR active sites.

(14) $${\mathrm{TOF}}_{\mathrm{ORR}}=3600\times 1.56\times 10^{15}\times A_{0}\times \frac{6-n}{2}\times \frac{\left|j_{\mathrm{ORR}}\right|}{\mathrm{ECSA}}\times h^{-1}=1471.4\times \frac{6-n}{2}\times \frac{\left|j_{\mathrm{ORR}}\right|}{\mathrm{ECSA}}\times h^{-1}$$

### 3.4. Electrochemical Performances of Assembled Rechargeable Zn–Air Batteries with NPCNSs-1000

**Figure 7:**
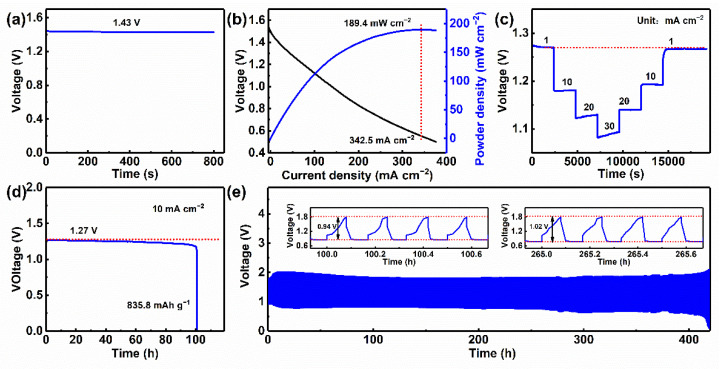
(**a**) Initial profile of open-circuit voltage, (**b**) discharge polarization curve and the power density profile, (**c**) rate performance from 1 to 30 mA cm^−2^, (**d**) continuous galvanostatic discharge curve at 10 mA cm^−2^, (**e**) galvanostatic discharge and charge cycling curve of Zn–air battery assembled with NPCNSs-1000 at 10 mA cm^−2^ with each cycle for 20 min (discharge for 10 min and charge for 10 min).

To evaluate the practical applicability of NPCNSs-1000, the electrochemical properties of the rechargeable liquid Zn−air batteries assembled with NPCNSs-1000 were measured and the results are shown in Figure 7. The Zn–air battery has a high open-circuit voltage of ~1.43 V (Figure 7a). A striking peak power density of ~189.4 mW cm^−2^ is obtained at a high current density of ~342.5 mA cm^−2^ (Figure 7b). There are no significant decreases in all discharge voltages, and the discharge voltages are nearly 100% recovered when the current densities return to the previous levels (Figure 7c), indicating an excellent rate of performance. Additionally, Zn–air battery derives the high voltage plateaus, i.e., ~1.27, ~1.18, ~1.13, and ~1.09 V at current densities of 1, 10, 20, and 30 mA cm^−2^, respectively. The Zn–air battery shows a high specific capacity of ~835.8 mAh g^−1^ at 10 mA cm^−2^ (Figure 7d), which is comparable and even superior to some recently reported catalysts (Table S6) [29,56,57,58,59,60,61,62]. Furthermore, the NPCNSs-1000 air cathode causes a subtle change in the discharge voltage after a continuous discharge for 100.3 h, suggesting the excellent durability of the NPCNSs-1000 air cathode. A smaller voltage gap of ~0.94 V is achieved at 100 h, and only a negligible loss of 0.08 V exists within 265 h (Figure 7e), indicating a good long-term cycling stability for the NPCNSs-1000 air cathode. Finally, the Zn–air batteries assembled with NPCNSs-1000 were out of service after 420 h.

## 4. Conclusions

In summary, we successfully prepared the NPCNSs via a one-step calcination/self-activation process of solvothermally synthesized 2D Zn-MOCs and calculated TOF_ORR_ taking into account the electron-transfer number (*n*). The trimodal pore structures, high specific surface area (*S*_BET_, ~1300.8 m^2^ g^−1^), a higher pore volume (*V*_total_, ~2.2 cm^3^ g^−1^), high-ratio mesopores (*V*_meso_/*V*_tota_, ~18.1%), massive vacancies (~5.8%)/long-range structural defects, and high-content pyridinic-N (~2.1%) highly favored for an enhanced ORR activity (*j*_s_, ~5.99 mA cm^−2^; *E*_1/2_, ~0.89 V; *b*, ~96.2 mV dec^−1^; TOF_ORR_, 23.0 h^−1^), a higher selectivity to four-electron ORR (~100%) and a superior stability ($r_{0.8}^{7500}$, ~82.4%; Δ*j*_s_, 0.58 mA cm^−2^). Additionally, the assembled Zn–air battery with NPCNSs-1000 derives a high peak power density of ~189.4 mW cm^−2^ at a high current density of ~342.5 mA cm^−2^, a high specific capacity of ~835.8 mAh g^−1^ at 10 mA cm^−2^ along with an excellent rate performance and stability. Furthermore, the experimental results prove that the enhancement of ORR activity mainly originates from defects and pyridinic-N. The above features adequately embody the great potential of the NPCNSs towards a durable air cathode for rechargeable Zn–air batteries.

## Data Availability

The data presented in this work are available on request from the corresponding authors.

## Supplementary Materials

The following supporting information can be downloaded at: https://www.mdpi.com/article/10.3390/polym14132581/s1, Figure S1: XRD patterns of Zn-MOCs-*x* (*x* = 0, 50, 100, 150, 200, and 300); Figure S2: CV curves of (a) NPCNSs-900 and (b) NPCNSs-1100 in the Ar-saturated and O_2_-saturated electrolyte solutions; Figure S3: (a) LSV curves and (b) RRDE curves for NPCNSs-1000; Figure S4: (a,d) LSV curves at the different scanning rates in the O_2_-saturated 0.1 M KOH solutions, (b,e) RRDE curves and (c,f) ${\mathrm{HO}}_{2}^{-}$ yield and electron-transfer number (*n*) for (a–c) NPCNSs-900 and (d–f) NPCNSs-1100; Figure S5: (a–c) CV curves at different scanning rates in the potential range from 1.066 to 1.166 V vs. RHE for (a) NPCNSs-900, (b) NPCNSs-1000, and (c) NPCNSs-1100; (d) the capacitive currents as a function of the scanning rates for NPCNSs-*y* (*y* = 900, 1000, and 1100); Figure S6: (a) Nyquist plots and the equivalent circuit diagram, (b) Bode plots of NPCNSs-900, NPCNSs-1000 and NPCNSs-1100; Figure S7: Bode plot of NPCNSs-1000 after the CP test; Table S1: Structural parameters derived from XRD patterns of NPCNSs-*y* (*y* = 900, 1000 and 1100); Table S2: The pore structure parameters of NPCNSs-*y* (*y* = 900, 1000, and 1100); Table S3: XPS peak positions, contents of C, N and O species in NPCNSs-*y* (*y* = 900, 1000, and 1100); Table S4: The fitted impedance values for Nyquist plots of NPCNSs-900, NPCNSs-1000, and NPCNSs-1100; Table S5: Comparison of ORR intrinsic activity between NPCNSs-1000 and recent reported electrocatalysts; Table S6: Comparison of electrocatalytic performances between NPCNSs-1000 and recent reported electrocatalysts applied in Zn–air batteries.
