# Supplementary material for: 2D Zinc-Based Metal–Organic Complexes Derived N-Doped Porous Carbon Nanosheets as Durable Air Cathode for Rechargeable Zn–Air Batteries
Source: Polymers (Basel). 2022 Jun 25;14(13):2581. doi: 10.3390/polym14132581 (PMC9269493; doi:10.3390/polym14132581)
Supplement: Supplementary file 1 [file polymers-14-02581-s001.zip › polymers-1763318-supplementary.pdf]

polymers MDPI

Supplemental Materials: 2D Zinc-Based Metal−Organic Complexes Derived N-doped Porous Carbon Nanosheets as Durable Air Cathode for Rechargeable Zn−Air Batteries

Peng Jia *, Jiawei Zhang, Guangmei Xia, Zhenjiang Yu, Jiazhen Sun and Xingxiang Ji *

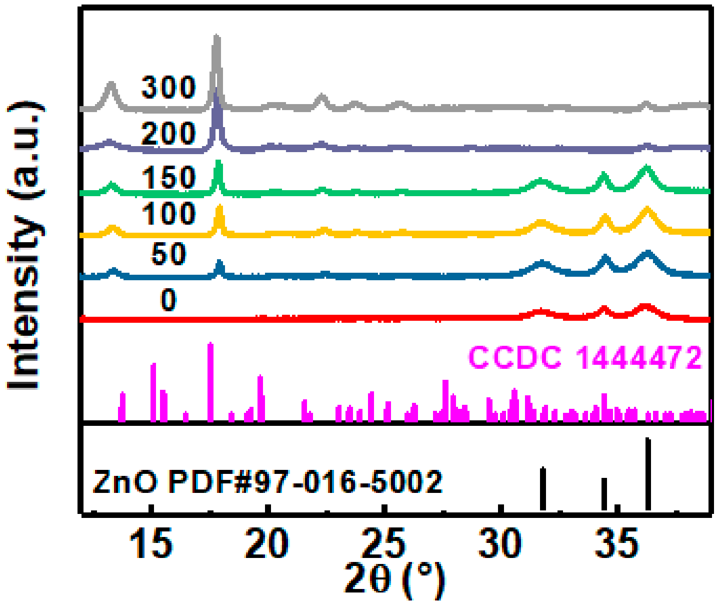

Figure S1. XRD patterns of Zn-MOCs-x (x = 0, 50, 100, 150, 200, and 300).

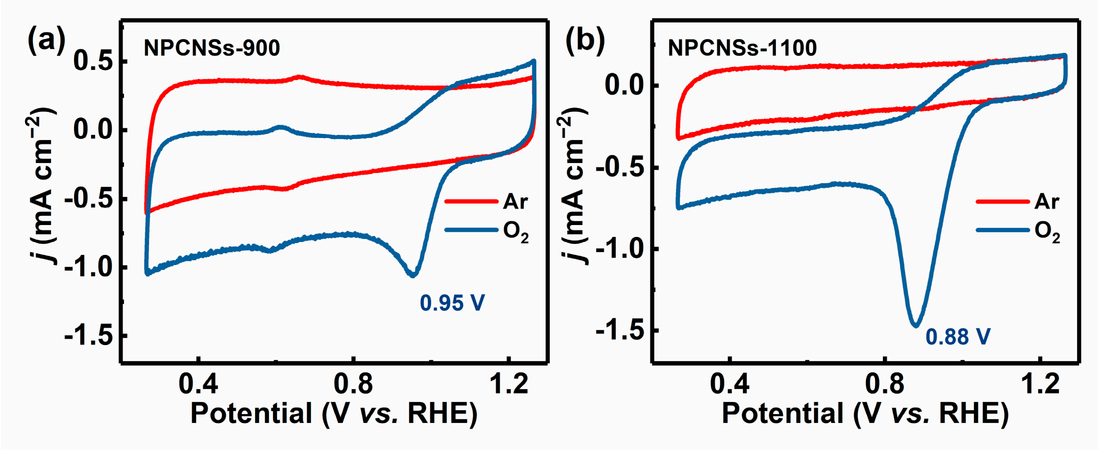

Figure S2. CV curves of (**a**) NPCNSs-900 and (**b**) NPCNSs-1100 in the Ar-saturated and O_2-saturated electrolyte solutions.

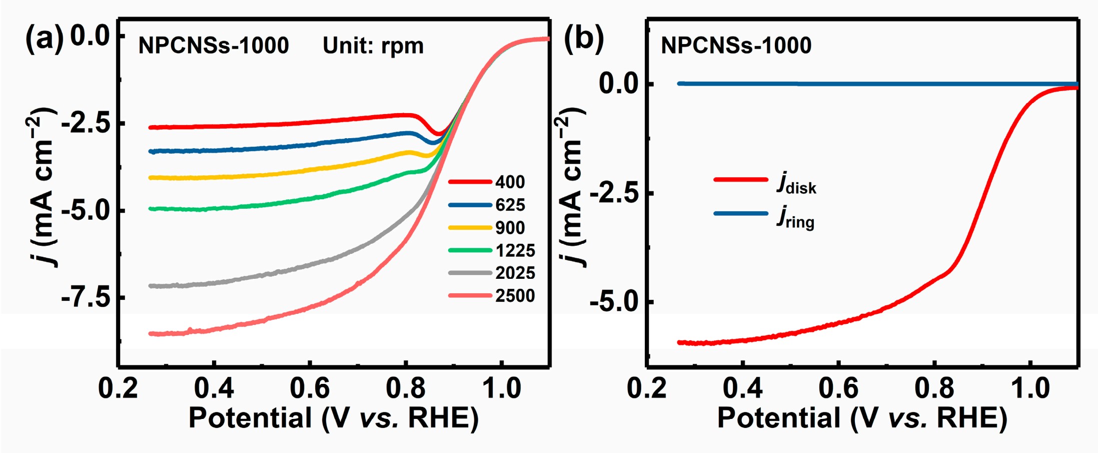

Figure S3. (**a**) LSV curves and (**b**) RRDE curves for NPCNSs-1000.

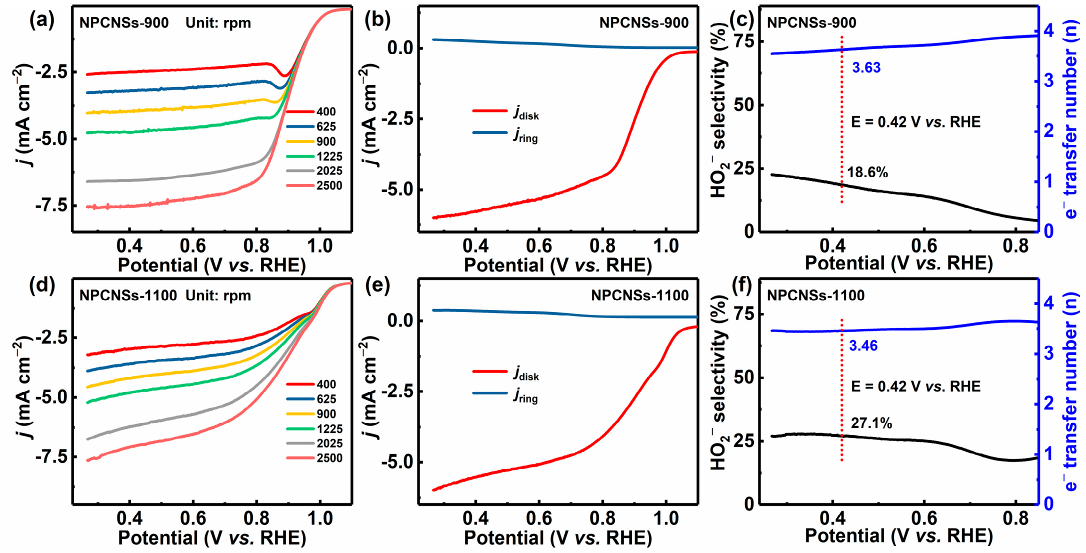

Figure S4. (**a**,**d**) LSV curves at the different scanning rates in the O2-saturated 0.1 M KOH solutions, (**b**,**e**) RRDE curves and (**c**,**f**) HO_2^- yield and electron-transfer number (*n*) for (**a**,**b**,**c**) NPCNSs-900 and (**d**,**e**,**f**) NPCNSs-1100.

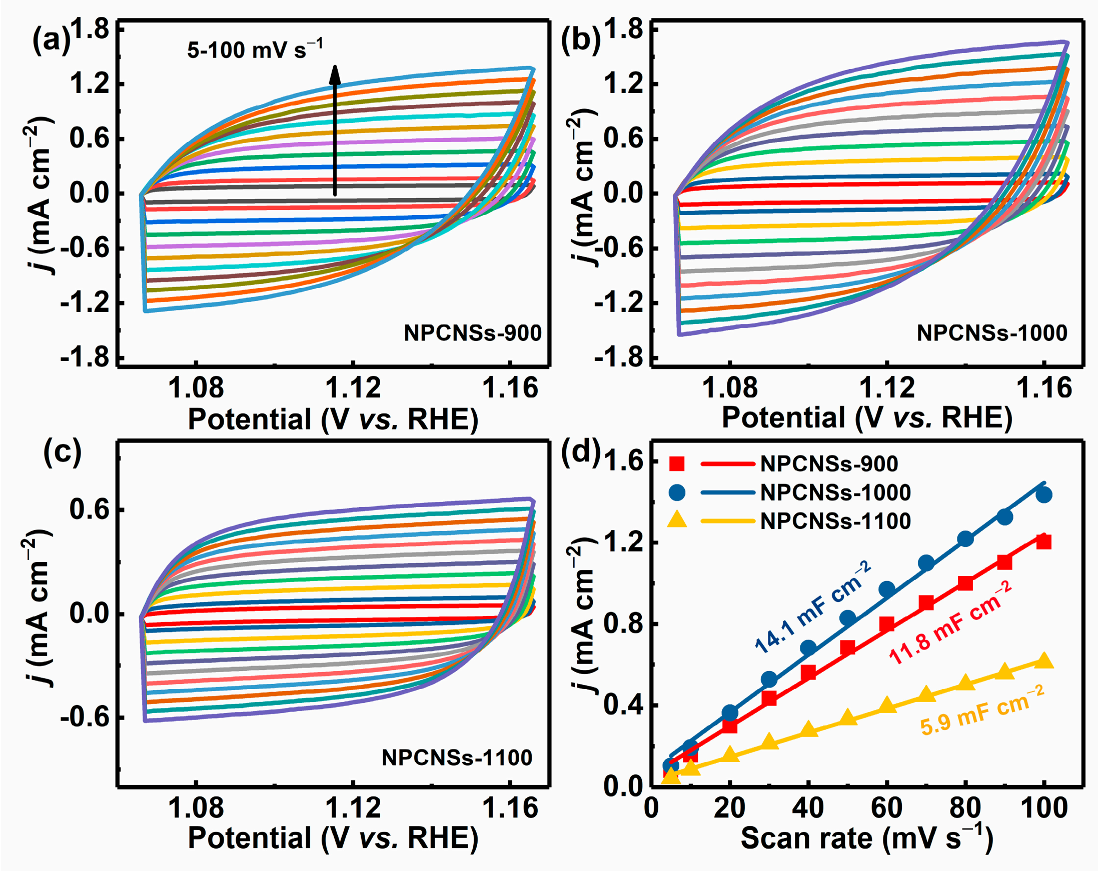

Figure S5. (**a**–**c**) CV curves at different scanning rates in the potential range from 1.066 to 1.166 V *vs*. RHE for (**a**) NPCNSs-900, (**b**) NPCNSs-1000 and (**c**) NPCNSs-1100; (**d**) the capacitive currents as a function of the scanning rates for NPCNSs-*y* (*y* = 900, 1000 and 1100).

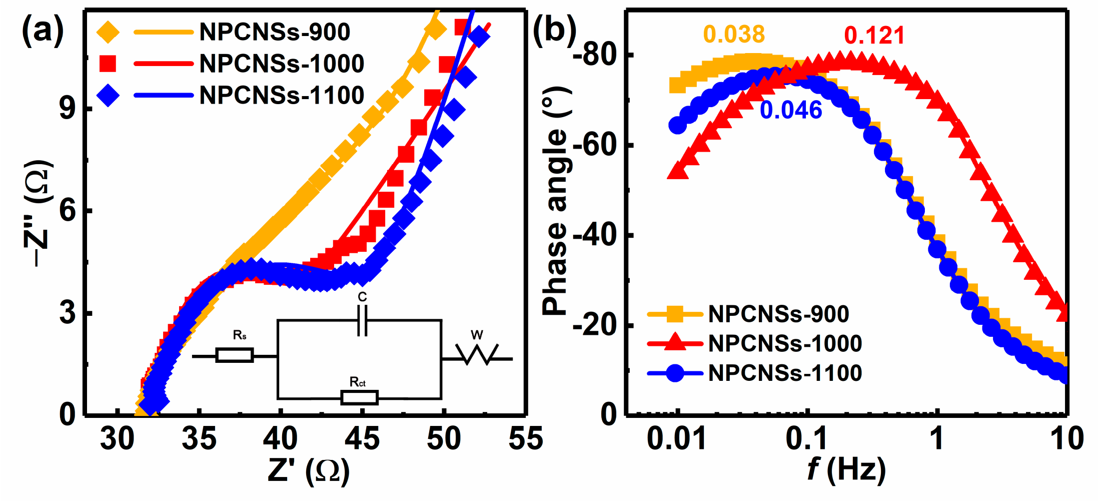

Figure S6. (**a**) Nyquist plots and the equivalent circuit diagram, (**b**) Bode plots of NPCNSs-900, NPCNSs-1000 and NPCNSs-1100.

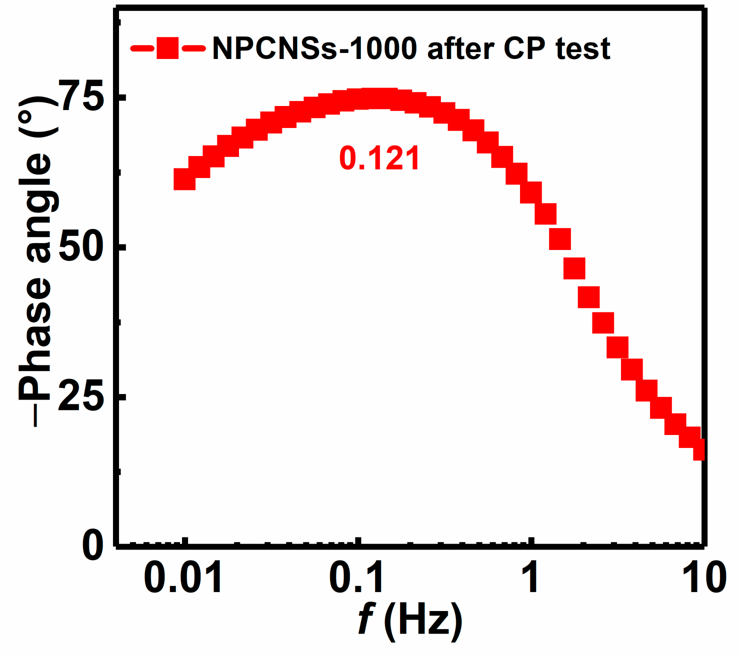

Figure S7. Bode plot of NPCNSs-1000 after the CP test.

Table S1. Structural parameters derived from XRD patterns of NPCNSs-*y* (*y* = 900, 1000 and 1100).

Sample	θ_{002}	θ_{100}	β_{002}	β_{100}	d_{002} (nm)	L_a (nm)	L_c (nm)
NPCNSs-900	12.53°	21.83°	0.209	0.109	0.355	2.80	0.66
NPCNSs-1000	12.50°	21.80°	0.235	0.115	0.356	2.67	0.59
NPCNSs-1100-	12.80°	21.85°	0.228	0.137	0.348	2.23	0.60

Table S2. The pore structure parameters of NPCNSs-*y* (*y* = 900, 1000 and 1100).

Sample	S_{BET} (m^2 g^{-1})	S_{micro} (m^2 g^{-1})	V_{total} (cm^3 g^{-1})	V_{micro} (cm^3 g^{-1})	V_{meso} (cm^3 g^{-1})	V_{micro}/ V_{total} (%)	V_{meso}/ V_{total} (%)	APD[a] (nm)
NPCNSs-900	942.5	714.8	1.7	1.5	0.2	88.2	11.8	6.7
NPCNSs-1000	1300.8	1122.8	2.2	1.8	0.4	81.8	18.1	7.3
NPCNSs-1100	1129.7	880.7	2.0	1.7	0.3	85.0	15.0	7.1

[a]Average pore diameter obtained by the BJH method.

Table S3. XPS peak positions, contents of C, N and O species in NPCNSs-y (y = 900, 1000 and 1100).

NPCNSs-900	Bing energy (eV)	Ratio (%)	NPCNSs-1000	Ra-tion (%)	NPCNSs-1100	Ration (%)
C (90.8%)			C (91.7%)		C (95.7%)	
C-I	284.1	3.2	C-I	5.8	C-I	4.2
C-II	284.7	33.5	C-II	31.3	C-II	40.8
C-III	285.1	33.3	C-III	32.1	C-III	31.6
C-IV	286.1	14.3	C-IV	17.2	C-IV	16.6
C-V	289.0	6.5	C-V	5.3	C-V	2.5
N (4.5%)			N (3.9%)		N (1.7%)	
N-I	398.4	0.8	N-I	0.5	N-I	0
N-II	400.3	1.2	N-II	2.1	N-II	0.2
N-III	401.4	2.5	N-III	1.3	N-III	1.5
O (4.7%)			O (4.4%)		O (2.6%)	
O-I	531.0	1.5	O-I	0.3	O-I	0.3
O-II	532.3	2.1	O-II	2.5	O-II	1.1
O-III	533.0	1.1	O-III	1.6	O-III	1.2

Table S4. The fitted impedance values for Nyquist plots of NPCNSs-900, NPCNSs-1000 and NPCNSs-1100.

Samples	R_s (Ω)	R_{ct} (Ω)
NPCNSs-900	32.1	20.0
NPCNSs-1000	31.8	10.6
NPCNSs-1100	32.0	14.5

Table S5. Comparison of ORR intrinsic activity between NPCNSs-1000 and recent reported electro-catalysts.

Catalyst	Electrolyte	TOF (h^{-1})
N-GRW [1]	0.1 M KOH	28.8
Fe-N-C [2]	0.5 M H_2SO_4	18.0
Co-N-C [3]	0.5 M H_2SO_4	36.0
Cu-N-C [4]	0.1 M $HClO_4$	5.4
$(Fe,Fe)_1 + N_2/H_2$ [5]	0.1 M $HClO_4$	54.0
NC [6]	0.1 M KOH	85.5
NPCNSs-1000 [This work]	0.1 M KOH	23.0

Table S6. Comparison of electrocatalytic performances between NPCNSs-1000 and recent reported electrocatalysts applied in Zn-Air batteries.

Catalyst	Electrolyte	Specific capacity (mAh g^{-1})	Power density (mW cm^{-2})
SA-Fe-NHPC [7]	6.0 M KOH + 0.2 M Zn $(Ac)_2$	/	266.4
ZOMC [8]	6.0 M KOH + 0.2 M Zn $(Ac)_2$	795.3	221.1
Cu-Fe@C [9]	6.0 M KOH	/	212.0
CoSA/N, S-HCS [10]	6.0 M KOH + 0.2 M Zn $(Ac)_2$	781.1	173.1
Co@NHCC-800 [11]	6.0 M KOH + 0.2 M Zn $(Ac)_2$	/	248.0
P, S-CNS [12]	6.0 M KOH	830.0	198.0
Co/$ZnCo_2O_4$@NC-CNTs [13]	6.0 M KOH + 0.2 M Zn $(Ac)_2$	922.0	305.0
Zn-NC/GD [14]	6.0 M KOH	824.7	326.1
NPCNSs-1000 [This work]	6.0 M KOH + 0.2 M Zn $(Ac)_2$	835.8	189.4

References

[1] Yang, H.; Miao, J.; Hung, S. F.; Chen, J.; Tao, H. B.; Wang, X.; Zhang, L.; Chen, R.; Gao, J.; Chen, H. M.; Dai, L.; Liu, B. Identification of catalytic sites for oxygen reduction and oxygen evolution in N-doped graphene materials: Development of highly efficient metal-free bifunctional electrocatalyst. *Sci. Adv.* **2016**, *2*, e1501122. DOI: https://doi.org/10.1126/sciadv.1501122

[2] Kramm, U. I.; Herrmann-Geppert. I.; Behrends, J.; Lips, K.; Fiechter, S.; Bogdanoff, P. On an easy way to prepare metal–nitrogen doped carbon with exclusive presence of MeN_4-type sites active for the ORR. *J. Am. Chem. Soc.* **2016**, *138*, 635–640. DOI: https://doi.org/10.1021/jacs.5b11015

[3] Chen, L.; Liu, X.; Zheng, L.; Li, Y.; Guo, X.; Wan, X.; Liu, Q., Shang, J.; Shui, J. Insights into the role of active site density in the fuel cell performance of Co-N-C catalysts. *Appl. Catal., B* **2019**, *256*, 117849. DOI: https://doi.org/10.1016/j.apcatb.2019.117849

[4] Luo, F.; Roy, A.; Silvioli, L.; Cullen, D. A.; Zitolo, A.; Sougrati, M. T.; Oguz, I. C.; Mineva, T.; Teschner, D.; Wagner, S.; Wen, J.; Dionigi, F.; Kramm, U. I.; Rossmeisl, J.; Jaouen, F.; Strasser, P. P-block single-metal-site tin/nitrogen-doped carbon fuel cell cathode catalyst for oxygen reduction reaction. *Nat. Mater.* **2020**, *19*, 1215–1223. DOI: https://doi.org/10.1038/s41563-020-0717-5

[5] Xiao, M.; Zhu, J.; Ma, L.; Jin, Z.; Ge, J.; Deng, X.; Hou, Y.; He, Q.; Li, J.; Jia, Q.; Mukerjee, S.; Yang, R.; Jiang, Z.; Su, D.; Liu, C.; Xing, W. Microporous framework induced synthesis of single-atom dispersed Fe-N-C acidic ORR catalyst and its in situ reduced $Fe-N_4$ active site identification revealed by x-ray absorption spectroscopy. *ACS Catal.* **2018**, *8*, 2824–2832 DOI: https://doi.org/10.1021/acscatal.8b00138

[6] Rybarczyk, M. K.; Gontarek, E.; Lieder, M.; Titirici, M. M. Salt melt synthesis of curved nitrogen-doped carbon nanostructures: ORR kinetics boost. *Appl. Surf. Sci.* **2018**, *435*, 543–551.DOI: https://doi.org/10.1016/j.apsusc.2017.11.064

[7] Chen, G.; Liu, P.; Liao, Z.; Sun, F.; He, Y.; Zhong, H.; Zhang, T.; Zschech, E.; Chen, M.; Wu, G.; Zhang, J.; Feng, X. Zinc-mediated template synthesis of Fe-N-C electrocatalysts with densely accessible Fe-N_x active sites for efficient oxygen reduction. *Adv. Mater.* **2020**, *32*, 1907399. DOI: https://doi.org/10.1002/adma.201907399

[8] Douka, A. I.; Xu, Y.; Yang, H.; Zaman, S.; Yan, Y.; Liu, H.; Salam, M. A.; Xia, B. Y. A zeolitic-imidazole frameworks-derived interconnected macroporous carbon matrix for efficient oxygen electrocatalysis in rechargeable Zinc-Air batteries. *Adv. Mater.* **2020**, *32*, 2002170. DOI: https://doi.org/10.1002/adma.202002170

[9] Nam, G.; Park, J.; Choi, M.; Oh, P.; Park, S.; Kim, M. G.; Park, N.; Cho, J.; Lee, J. S. Carbon-coated core-shell Fe-Cu nanoparticles as highly active and durable electrocatalysts for a Zn-air battery. *ACS Nano* **2015**, *9*, 6493−6501. DOI: https://doi.org/10.1021/acsnano.5b02266

[10]Zhang, Z.; Zhao, X.; Xi, S.; Zhang, L.; Chen, Z.; Zeng, Z.; Huang, M.; Yang, H.; Liu, B.; Pennycook, S. J.; Chen, P. Atomically dispersed cobalt trifunctional electrocatalysts with tailored coordination environment for flexible rechargeable Zn-air battery and self-driven water splitting. *Adv. Energy Mater.* **2020**, *10*, 2002896. DOI: https://doi.org/10.1002/aenm.202002896

[11] Wu, J.; Hu, L.; Wang, N.; Li, Y.; Zhao, D.; Li, L.; Peng, X.; Cui, Z.; Ma, L. J.; Tian, Y.; Wang, X. Surface confinement assisted synthesis of nitrogen-rich hollow carbon cages with Co nanoparticles as breathable electrodes for Zn-air batteries. *Appl. Catal., B* **2019**, *254*, 55–65. DOI: https://doi.org/10.1016/j.apcatb.2019.04.064

[12] Shinde, S. S.; Lee, C. H.; Sami, A.; Kim, D. H.; Lee, S. U.; Lee, J. H. Scalable 3-D carbon nitride sponge as an efficient metal-free bifunctional oxygen electrocatalyst for rechargeable Zn-Air batteries. *ACS Nano* **2017**, *11*, 347−357. DOI: https://doi.org/10.1021/acsnano.6b05914

[13] Yan, L.; Xu, Z.; Hu, W.; Ning, J.; Zhong, Y.; Hu, Y. Formation of sandwiched leaf-like CNTs-Co/ZnCo2O4@NC-CNTs nanohybrids for high-power-density rechargeable Zn-Air batteries. *Nano Energy* **2021**, *82*, 105710. DOI: https://doi.org/10.1016/j.nanoen.2020.105710

[14] Deng, W.; Li, G.; Wu, T.; He, L.; Wu, J.; Liu, J.; Zheng, H.; Li, X.; Yang, Y.; Jing, M.; Wang, Y.; Wang, X. Heteroatom functionalized double-layer carbon nanocages as highly efficient oxygen electrocatalyst for Zn-Air batteries. *Carbon.* **2022**, *186*, 589–598. DOI: https://doi.org/10.1016/j.carbon.2021.10.057
